# Changing nationwide trends away from overtreatment among patients undergoing radical prostatectomy over the past 25 years

**DOI:** 10.1007/s00345-023-04418-8

**Published:** 2023-05-17

**Authors:** Valentin H. Meissner, Viviane Glöckler, Matthias Jahnen, Stefan Schiele, Jürgen E. Gschwend, Kathleen Herkommer

**Affiliations:** Department of Urology, Klinikum rechts der Isar, School of Medicine, Technical University of Munich, Ismaninger Strasse 22, 81675 Munich, Germany

**Keywords:** Prostate cancer, Radical prostatectomy, Active surveillance, Overtreatment, Risk classification, Treatment trends

## Abstract

**Purpose:**

The objective of the current study was to assess whether and how preoperative risk group distribution and pathological outcomes have changed in men treated with radical prostatectomy (RP) over the past 25 years.

**Methods:**

11,071 patients from a large contemporary registry-based nationwide cohort with RP as primary treatment between 1995 and 2019 were included. Preoperative risk stratification, postoperative outcomes, and 10 years other-cause mortality (OCM) were analyzed.

**Results:**

After 2005, the proportion of low-risk prostate cancer (PCa) decreased from 39.6% to 25.5% in 2010 and decreased further to 15.5% in 2015, and 9.4% in 2019 (*p* < 0.001). The proportion of high-risk cases increased from 13.1% in 2005 to 23.1% in 2010 and 36.7% in 2015, and 40.4% in 2019 (*p* < 0.001). After 2005, the proportion of cases with favorable localized PCa decreased from 37.3% to 24.9% in 2010 and decreased further to 13.9% in 2015, and 1.6% in 2019 (*p* < 0.001). The overall 10 years OCM was 7.7%.

**Conclusion:**

The current analysis documents a clear shift in utilization of RP toward higher-risk PCa in men with long life expectancy. Patients with low-risk PCa or favorable localized PCa are rarely operated. This suggests a shift in applying surgery only to patients who may really benefit from RP and the long-standing discussion of overtreatment might become outdated.

**Supplementary Information:**

The online version contains supplementary material available at 10.1007/s00345-023-04418-8.

## Introduction

Radical prostatectomy (RP) remains one of the most commonly used first-line treatments for patients with clinically localized prostate cancer (PCa) [1]. However, the benefit concerning oncological outcome of RP is highly dependent on patient- and tumor-specific characteristics [2]. In patients at low-risk RP has no impact on overall or cancer-specific survival, whereas in men with intermediate or high-risk disease, RP significantly decreases mortality due to PCa [3, 4].

The advent of prostate-specific antigen (PSA) testing and its use for early detection and screening for PCa increased stage migration toward more favorable stages [5]. However, substantial rates of overdiagnosis and subsequent overtreatment have been identified as major downsides of PSA testing. Therefore, active surveillance (AS), with the intent to initiate definitive treatment if there is evidence of disease progression, is currently the preferential initial management strategy in low-risk PCa to reduce overtreatment [6]. Conversely, patients with high-risk diseases appear to benefit the most from definitive therapy [7]. About 40% of high-risk patients have organ-confined disease following RP with excellent long-term outcomes while avoiding long-term androgen deprivation therapy [8].

In the last decade, the risk profile of patients showed an inverse trend towards more high-risk disease in RP-treated patients [9, 10]. This is related to multiple evolving factors such as the introduction of AS for low-risk PCa as well as the increased use of RP in multidisciplinary treatment approaches complemented by radiation, androgen deprivation, or chemotherapy in locally advanced or metastatic PCa patients [11–14].

The aim of the current study was to assess whether and how preoperative risk group distribution and pathological outcomes have changed in men treated with RP over the past 25 years based on above-mentioned developments in a large contemporary registry-based nationwide cohort.

## Patients and methods

Data for the current analysis were obtained from the nationwide multicenter German Familial Prostate Cancer study and its database [15, 16]. Since 1994, PCa patients have been prospectively recruited by collaborating clinics and urologists throughout Germany. The database comprises more than 40,000 PCa patients and collects a range of sociodemographic, diagnostic, clinicopathological, and treatment outcome data on PCa patients in Germany. Patient information are updated annually via questionnaires. Informed consent is obtained from each patient. The study was reviewed and approved by the ethical review committee of the Technical University of Munich. For the current analysis, patients were eligible if they had RP between January 1995 and December 2019. Patients after salvage RP were excluded.

Patient characteristics were stratified by year of RP. Clinicopathological characteristics included age at RP, prostate-specific antigen (PSA) level at diagnosis, neoadjuvant or adjuvant therapy, clinical and pathological TNM classification, Gleason Grade Group (GG) of biopsy and RP specimen, and surgical margin. Pathological staging was classified or reclassified for patients diagnosed before 2002 using the UICC TNM classification 2002. Preoperative risk group distribution was applied according to the current European Association of Urology (EAU) guidelines [17]. Following precedents in the literature [13], the definition of favorable localized* PCa* (defined as ≤ pT2c disease, Gleason GG 1, pN0/X, cM0/X, no adjuvant or neoadjuvant therapy, and PSA ≤ 20 ng/ml) was used as a surrogate to label the patients that are most likely to not have benefitted of RP and likely to have been overtreated, at least from an oncological point of view [13]. Additionally to suitable cancer selection, adequate patient selection based on age, comorbidities, and life expectancy is the other instrument used to avoid overtreatment and can be estimated by the rate of other-cause mortality (OCM) in the RP series.

Data analyses were conducted using the Statistical Analysis System (SAS), version 9.4 (SAS Institute Inc., Cary, NC, USA). Descriptive statistics were used to present participant characteristics. Comparison of risk group distribution and a fraction of favorable localized PCa between years of surgery were conducted using chi-square tests. Differences in age at surgery over the years were examined with linear regression for each risk group separately. *p* values < 0.05 were considered statistically significant (2-sided test).

## Results

Table 1 presents clinical and pathological patient characteristics as well as risk group distribution of the 11,071 patients included in the study. In general, the cohort is typical of patients undergoing RP in Germany over the last decades. The overall 10 years OCM was 7.7% and men < 65 years at diagnosis had a 10 years OCM of 5.0%.

**Table 1:** Mean stable isotope ratios values and standard error for squamosal, rib, and epidermis of green turtles from Oahu and Kona/Kohala coast

Characteristics	
Age at radical prostatectomy, median (IQR), year	65.1 (60.4–69.4)
Positive PCa family history, *n* (%)	2725 (24.6)
PSA at diagnosis, median (IQR), ng/ml	7.5 (5.2–11.9)
Preoperative tumor stage, *n* (%)	
≤ cT1c	4685 (42.3)
cT2	6026 (54.5)
cT3	347 (3.1)
cT4	13 (0.1)
Clinical metastasis stage, *n* (%)	
cM0/X	10,995 (99.3)
cM1	70 (0.6)
Missing	6 (0.1)
Biopsy gleason grade group, *n* (%)	
1	5657 (51.1)
2	2219 (20.0)
2/3	504 (4.6)
3	1108 (10.0)
4	954 (8.6)
5	629 (5.7)
Clinical risk group distribution, *n* (%)	
Low risk	2875 (26.0)
Intermediate risk	5316 (48.0)
High risk	2440 (22.0)
Missing	440 (4.0)
Neoadjuvant therapy, *n* (%)	
Androgen deprivation therapy	575 (5.2)
Chemotherapy	94 (0.9)
Pathological tumor stage, *n* (%)	
≤ pT2c	7435 (67.2)
≥ pT3a	3607 (32.5)
Missing	29 (0.3)
Pathological node stage, *n* (%)	
pN0/X	10,014 (90.4)
pN1	1051 (9.5)
Missing	6 (0.1)
Surgical margin, *n* (%)	
R0	8,189 (74.0)
RX	184 (1.6)
R1	1805 (16.3)
Missing	893 (8.1)
Pathological Gleason Grade Group, *n* (%)	
1	4015 (36.3)
2	3074 (27.8)
2/3	618 (5.6)
3	1480 (13.4)
4	737 (6.6)
5	754 (6.8)
Missing	393 (3.5)
Favorable localized PCa^a^, *n* (%)	3069 (28.3)
Missing	215 (1.9)
Adjuvant therapy, *n* (%)	
Radiatio	872 (7.9)
Androgen deprivation therapy	618 (5.6)
Chemotherapy	7 (0.1)
10 years other cause mortality, *n* (%)	410 (7.7)
< 65 years	138 (5.0)
≥ 65 years	272 (10.6)
10 years cancer-specific survival, %	
1995–1999	
Low risk	98.3
Intermediate risk	97.1
High risk	84.9
2000–2004	
Low risk	99.1
Intermediate risk	96.7
High risk	88.6
2005–2009	
Low risk	99.6
Intermediate risk	97.3
High risk	85.9

*IQR* inter-quartile range, *PCa* prostate cancer, *PSA* prostate-specific antigen

^a^Defined as ≤ pT2c disease, Gleason Grade Group 1, pN0/X, cM0/X, PSA ≤ 20 ng/ml

**Fig. 1:**
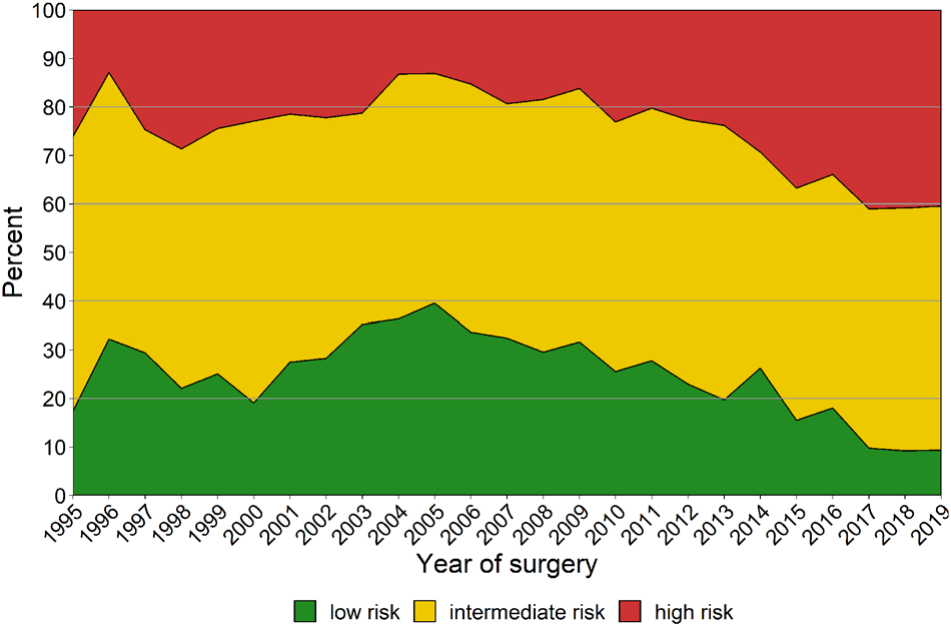
Preoperative risk group distribution according to the European Association of Urology guidelines [17], per year of surgery

Figure 1 presents the risk group distribution according to EAU guidelines, per year of surgery. After 2005, the proportion of low-risk PCa decreased from 39.6 to 25.5% in 2010 and decreased further to 15.5% in 2015, and 9.4% in 2019 (*p* < 0.001). In the same period, the proportion of cases considered high-risk PCa increased from 13.1% in 2005 to 23.1% in 2010 and 36.7% in 2015 and 40.4% in 2019 (*p* < 0.001).

**Fig. 2:**
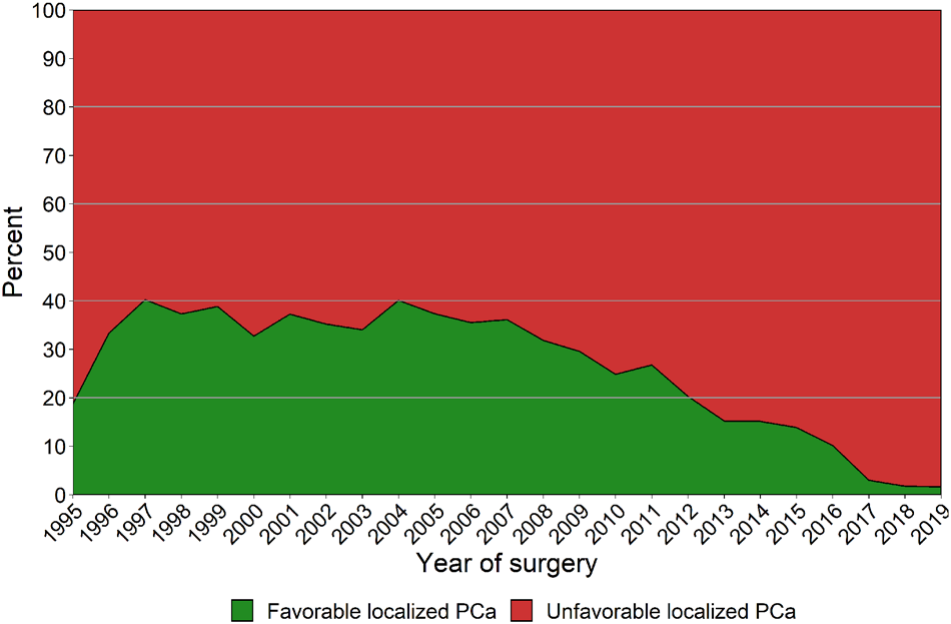
Postoperative distribution of favorable localized PCa (≤ pT2c disease, Gleason Grade Group 1, pN0/X, cM0/X, no adjuvant or neoadjuvant therapy, and PSA ≤ 20 ng/ml) [13], per year of surgery

Figure 2 presents the clinicopathological distribution of men with favorable localized PCa (≤ pT2c, Gleason GG 1, pN0/X, cM0/X, no adjuvant or neoadjuvant therapy, and PSA ≤ 20 ng/ml) after RP, per year of surgery. After 2005, the proportion of cases with favorable localized PCa decreased from 37.3 to 24.9% in 2010 and decreased further to 13.9% in 2015, and 1.6% in 2019 (*p* < 0.001).

The abovementioned trends were confirmed in further analyses of PSA levels, tumor stage, and Gleason GG, respectively, per year of surgery (Supplementary Figs. 1, 2, 3).

**Fig. 3:**
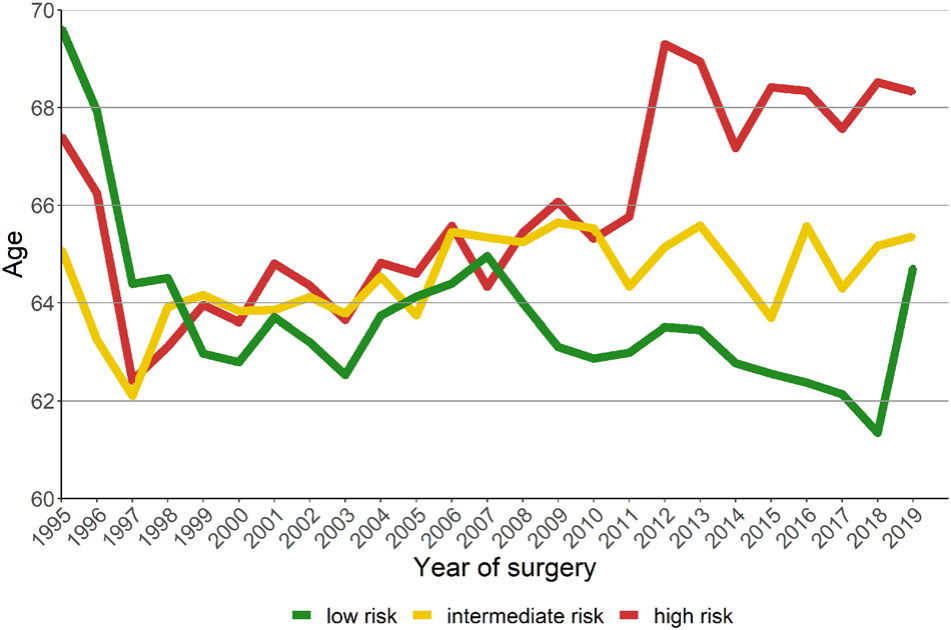
Median age of high-risk, intermediate-risk, and low-risk PCa patients, per year of surgery

The median age of high, intermediate, and low-risk PCa patients, per year of surgery, is depicted in Fig. 3. While the age of low-risk PCa patients remained stable (*p* = 0.064), it increased in intermediate and high-risk patients (both *p* < 0.001). Men who chose RP as the primary treatment of low-risk PCa were younger than RP candidates with higher risk profiles (Fig. 3).

## Discussion

The current study reports contemporary RP outcome data of a nationwide registry-based cohort to analyze trends in risk group distribution and oncological outcomes of patients undergoing surgery for PCa in Germany.

Main results showed an important decrease in patients with preoperative low-risk PCa after 2005 (from more than 1 in 3 patients to 1 in 10 patients) of all patients. At the same time, the proportion of high-risk PCa patients increased from around 1 in 10 patients to more than 1 in 3. Further, the number of patients with favorable localized PCa in the final histopathological RP specimen, used as a surrogate to label the patients that are most likely to not have benefitted from RP and therefore oncologically may be considered overtreated, decreased from more than 1 in 3 to even less than 1 in 20 patients. The reason for such changes in risk group distribution and inverse stage and grade migration can only be hypothesized since patient selection is mostly done in the outpatient sector. Especially the advent of magnetic resonance imaging (MRI) and subsequent performed MRI-targeted biopsies in later years might have contributed substantially to stage and grade migration and might be one reason for the decreasing share of low-risk PCa patients. In both PROMIS and PRECISION, the detection rate of clinically insignificant PCa was lower in MRI-targeted biopsies compared to standard biopsies, whereas clinically significant cancers were more often detected [18, 19]. Another reason for the decreasing share of low-risk PCa patients could be the increasing use and acceptance of AS developed among urologists, since active therapy may be considered overtreatment in some low-risk PCa cases and several guidelines including the EAU guidelines strongly recommend the use of AS in low-risk PCa [17]. Indeed, the first reports on AS from the European Randomized Study of Screening for Prostate Cancer and initiation of the Prostate Cancer Research International Active Surveillance (PRIAS) study stem from 2006 to 2007 [20, 21]. The decreasing share among patients having favorable localized PCa who still underwent RP is primarily due to a shift away from GGG 1, whereas pathological tumor stage and PSA levels remained stable within years (see Supplementary Figs. 1, 2, 3). This suggests also an increased use of MRI-targeted biopsies and a better application of AS and risk-driven decisions between patients and urologists. The increase in the use of AS from 57 to 91% for very low-risk PCa and from 40 to 74% for low-risk PCa from 2009 to 2014, respectively, was reported in a population-based study in Sweden [23]. A further Australian study showed an increase in the use of AS from 24 to 39% from 2009 to 2012 [24]. On the other hand, the increasing share of higher-risk cases undergoing surgery may be due to improvements in surgical treatment options and the increasing use of RP in multidisciplinary treatment approaches complemented by radiation, androgen deprivation, or chemotherapy [11, 12, 14, 22].

While the results of the current study assume an evolution in the increasing use of AS in patients with favorable PCa disease characteristics in the last 25 years throughout Germany, the use of other treatments was not considered in the current analysis. However, other treatments such as radiotherapy have also been changing in recent years. For instance, the use of brachytherapy in low-risk PCa decreased over the past decades [25, 26].

A similar analysis in a large German high-volume center cohort was already performed in 2015 (Martini Klinik, Hamburg). In their brief correspondence, the authors encouraged other centers to do similar analyses [27]. Their findings were later confirmed on a European level, however, these results were likewise limited to high-volume center data [13]. The current study additionally presents contemporary data of a representative cross-section of patients in Germany not only limiting to high-volume center patients, since data were derived from high and low volume center, rehabilitation clinics, and primary care urologists throughout Germany.

There are several possibilities to avoid overtreatment such as suitable cancer selection as well as adequate patient selection based on age, performance status, comorbidities, and life expectancy. Adequate patient selection can be estimated by a low rate of OCM in surgical series. The overall 10 years OCM was 7.7% in this population. For instance, the OCM rate in the Prostate Cancer Intervention Versus Observation Trial (PIVOT) at the same point in time was 40% in both the control group and the surgical group [3]. However, these findings cannot be extrapolated to current clinical practice, since the patient selection was obviously inadequate in this cohort and might be, therefore, misleading. Conversely, the aforementioned German single-center study reported a lower rate of OCM compared to PIVOT. After a follow-up of 15 years, the overall OCM rate was low at 14.8%. This supports the findings of the current study and shows that contemporary patient selection is more than appropriate and has the potential to avoid overtreatment. Increasing life expectancy and consideration of comorbidities have the potential to further decrease overtreatment [28].

Strengths of the current analysis include the heterogeneity and the representative cross-section of the patient population as well as the large patient numbers, providing a good overview of the developments over the past 25 years. However, the use of other treatments was not assessed and risk group distribution within the current RP database only provides indirect evidence and no causal conclusions can be drawn from the observed results. For instance, patients might have chosen radiotherapy instead of surgery, which could lead to an overestimation of the reduction of definitive therapy in AS candidates. In addition, changes in diagnostics or treatment possibilities cannot be excluded as further potential causes of changes in risk group distribution.

## Conclusions

In the current analysis, a clear shift in the utilization of surgery toward high-risk PCa in men with long life expectancy is shown, whereas patients with low-risk PCa or favorable localized PCa are rarely operated. This confirms a shift in applying RP primarily to patients who may really benefit oncologically, whereas side effects are reduced in patients with the most favorable disease spectrum. Based on these developments, the long-standing discussion of overtreatment with RP might become outdated.

## Supplementary Information

Below is the link to the electronic supplementary material.

Supplementary file1 (PNG 94 KB) PSA level of the study sample, per year of surgery

Supplementary file2 (PNG 76 KB) Postoperative distribution of pathological tumor stage, per year of surgery

Supplementary file3 (PNG 109 KB) Postoperative distribution of Gleason Grade Group (GGG) of the radical prostatectomy specimen, per year of surgery

Supplementary file4 (DOCX 21 KB)

## Data Availability

Not applicable.
