# Supplementary figures and images for: Changing nationwide trends away from overtreatment among patients undergoing radical prostatectomy over the past 25 years
Source: World J Urol. 2023 May 17;41(6):1497–502. doi: 10.1007/s00345-023-04418-8 (PMC10241676; doi:10.1007/s00345-023-04418-8)

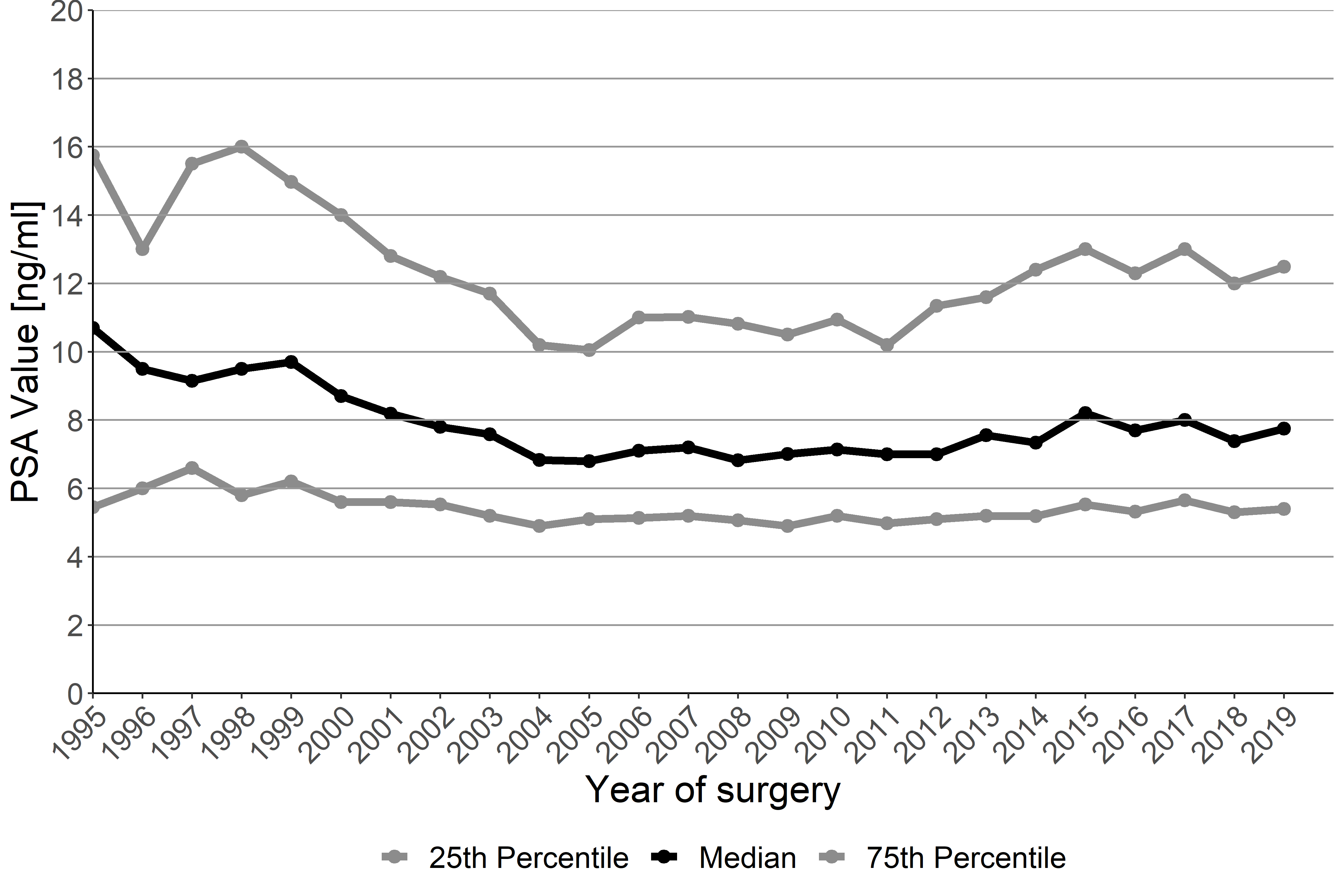

Supplement: Supplementary file 1 — Supplementary file1 (PNG 94 KB) PSA level of the study sample, per year of surgery [file 345_2023_4418_MOESM1_ESM.png]

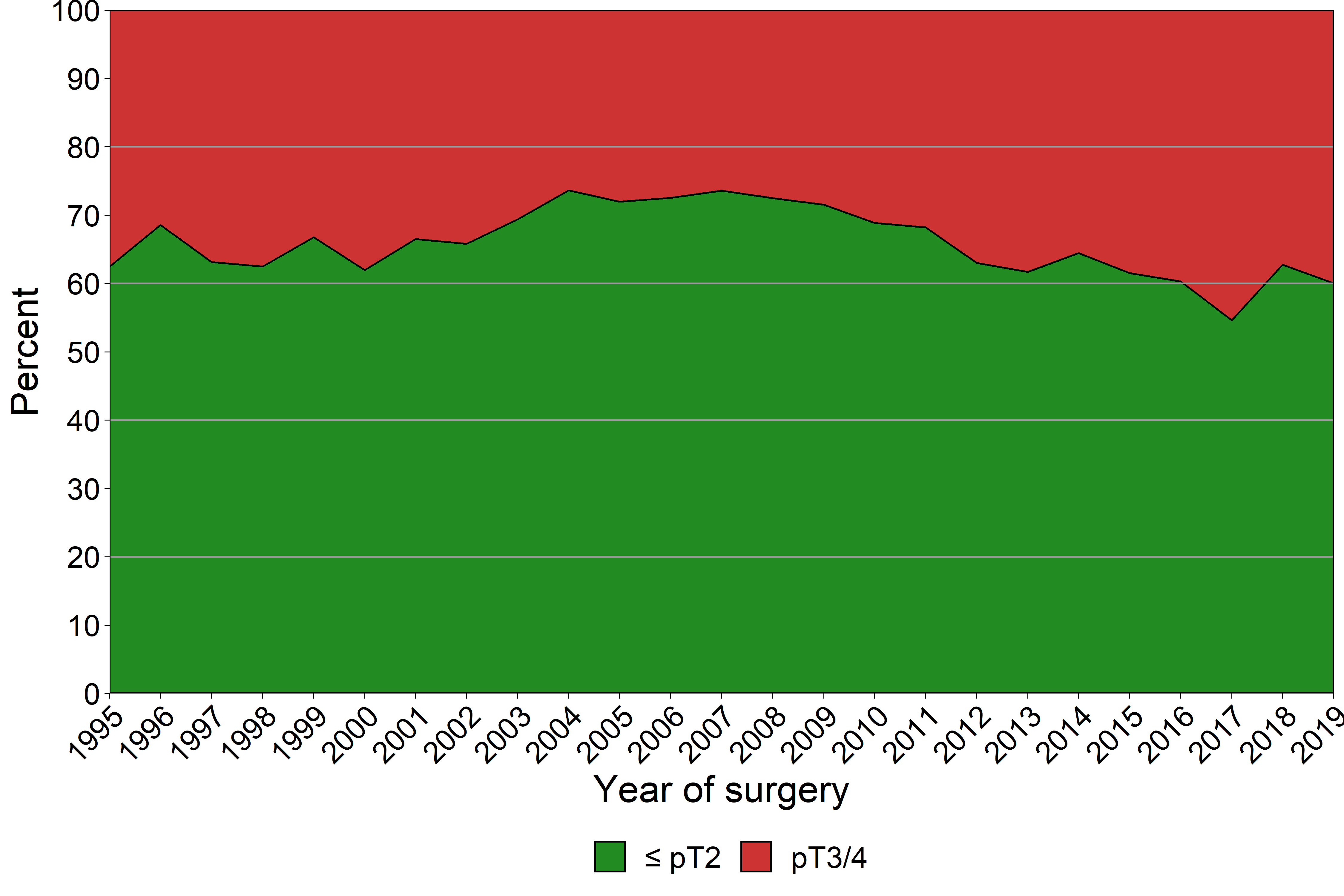

Supplement: Supplementary file 2 — Supplementary file2 (PNG 76 KB) Postoperative distribution of pathological tumor stage, per year of surgery [file 345_2023_4418_MOESM2_ESM.png]

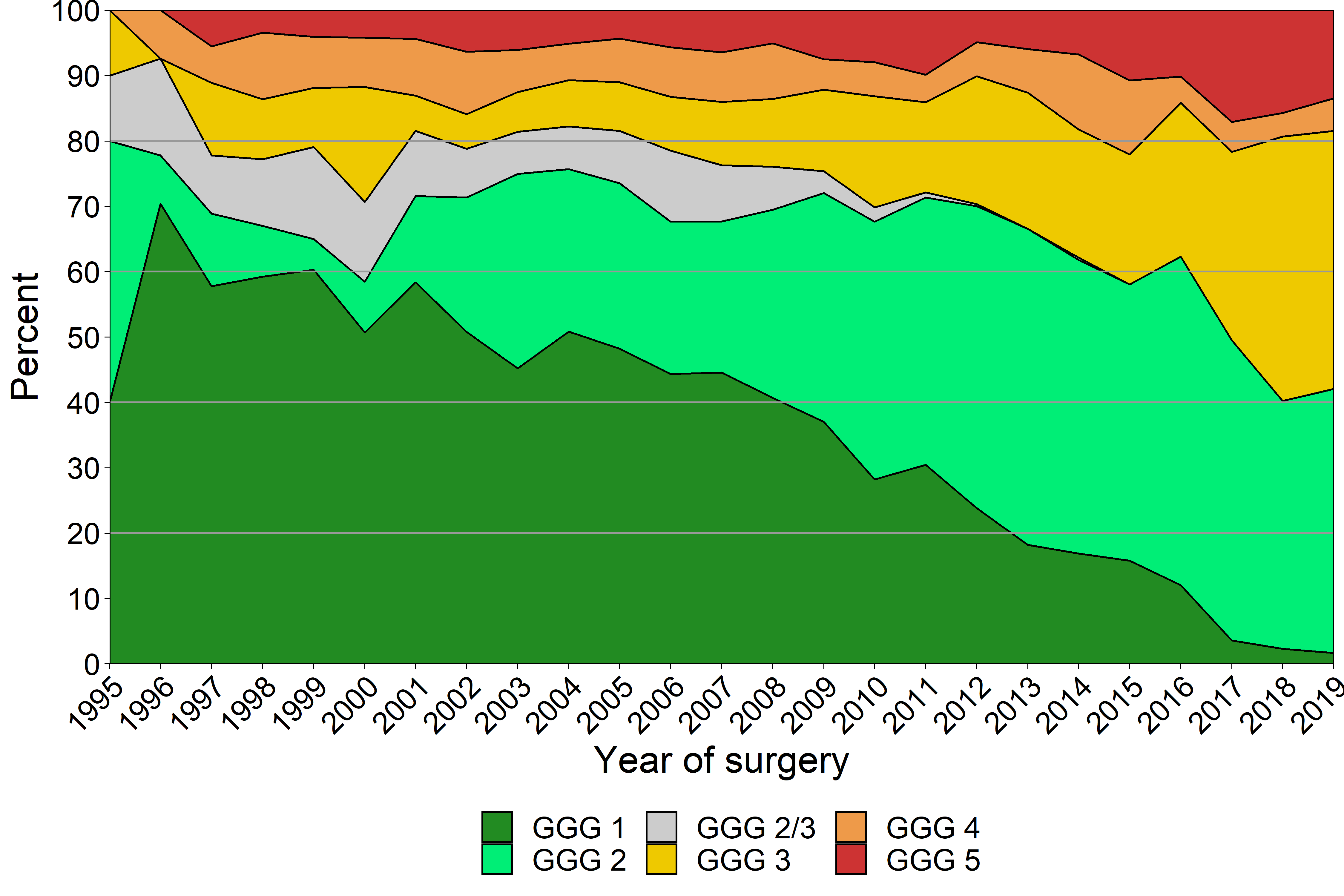

Supplement: Supplementary file 3 — Supplementary file3 (PNG 109 KB) Postoperative distribution of Gleason Grade Group (GGG) of the radical prostatectomy specimen, per year of surgery [file 345_2023_4418_MOESM3_ESM.png]
