# Supplementary material for: Changing nationwide trends away from overtreatment among patients undergoing radical prostatectomy over the past 25 years
Source: World J Urol. 2023 May 17;41(6):1497–502. doi: 10.1007/s00345-023-04418-8 (PMC10241676; doi:10.1007/s00345-023-04418-8)
Supplement: Supplementary file 4 — Supplementary file4 (DOCX 21 KB) [file 345_2023_4418_MOESM4_ESM.docx]

| **Supplementary Table 1.** Corresponding absolute numbers (%) of proportions given in Figure 1, Figure 2, Supplementary Figure 2, and Supplementary Figure 3 | | | | | | | | | | | | | | |
| --- | --- | --- | --- | --- | --- | --- | --- | --- | --- | --- | --- | --- | --- | --- |
|  |  | Total | 1995 | 1996 | 1997 | 1998 | 1999 | 2000 | 2001 | 2002 | 2003 | 2004 | 2005 | 2006 |
| Risk group distribution | |  |  |  |  |  |  |  |  |  |  |  |  |  |
|  | Low risk | 2875 | 4 (17.4) | 10 (32.3) | 25 (29.4) | 44 (22.1) | 77 (25.1) | 75 (19.1) | 182 (27.5) | 206 (28.3) | 206 (35.3) | 305 (36.4) | 369 (39.6) | 245 (33.7) |
|  | Intermediate risk | 5316 | 13 (56.5) | 17 (54.8) | 39 (45.9) | 98 (49.3) | 155 (50.5) | 228 (58.0) | 338 (51.1) | 361 (49.5) | 254 (43.5) | 423 (50.4) | 441 (47.3) | 372 (51.1) |
|  | High risk | 2440 | 6 (26.1) | 4 (12.9) | 21 (24.7) | 57 (28.6) | 75 (24.4) | 90 (22.9) | 142 (21.4) | 162 (22.2) | 124 (21.2) | 111 (13.2) | 122 (13.1) | 111 (15.2) |
| Favorable localized PCa | | 3069 | 3 (18.8) | 10 (33.3) | 37 (40.2) | 78 (37.3) | 127 (38.8) | 144 (32.7) | 271 (37.3) | 274 (35.2) | 200 (34.0) | 339 (40.1) | 348 (37.3) | 261 (35.5) |
| Tumor stage at RP (≤pT2) | | 7435 | 15 (62.5) | 24 (68.6) | 60 (63.2) | 135 (62.5) | 227 (66.8) | 280 (62.0) | 510 (66.5) | 535 (65.8) | 417 (69.4) | 643 (73.7) | 680 (72.0) | 534 (72.6) |
| GGG of RP specimen | |  |  |  |  |  |  |  |  |  |  |  |  |  |
|  | GGG 1 | 4015 | 4 (40.0) | 19 (70.4) | 52 (57.8) | 122 (59.2) | 193 (60.3) | 216 (50.7) | 411 (58.4) | 383 (50.8) | 260 (45.2) | 418 (50.8) | 446 (48.2) | 322 (44.3) |
|  | GGG 2 | 3074 | 4 (40.0) | 2 (7.4) | 10 (11.1) | 16 (7.8) | 15 (4.7) | 33 (7.8) | 93 (13.2) | 155 (20.6) | 171 (29.8) | 204 (24.8) | 234 (25.3) | 169 (23.3) |
|  | GGG 2/3 | 618 | 1 (10.0) | 4 (14.8) | 8 (9.0) | 21 (10.2) | 45 (14.1) | 52 (12.2) | 70 (9.9) | 56 (7.4) | 37 (6.4) | 54 (6.6) | 74 (8.0) | 79 (10.9) |
|  | GGG 3 | 1480 | 1 (10.0) | 0 | 10 (11.1) | 19 (9.2) | 29 (9.1) | 75 (17.6) | 38 (5.4) | 40 (5.3) | 35 (6.1) | 58 (7.1) | 69 (7.5) | 60 (8.3) |
|  | GGG 4 | 737 | 0 | 2 (7.4) | 5 (5.5) | 21 (10.2) | 25 (7.8) | 32 (7.5) | 61 (8.7) | 72 (9.6) | 37 (6.4) | 46 (5.6) | 62 (6.7) | 55 (7.6) |
|  | GGG 5 | 754 | 0 | 0 | 5 (5.5) | 7 (3.4) | 13 (4.0) | 18 (4.2) | 31 (4.4) | 48 (6.3) | 35 (6.1) | 42 (5.1) | 40 (4.3) | 41 (5.6) |
|  | | | | | | | | | | | | | | |
|  | | | | | | | | | | | | | | |
|  |  | 2007 | 2008 | 2009 | 2010 | 2011 | 2012 | 2013 | 2014 | 2015 | 2016 | 2017 | 2018 | 2019 |
| Risk group distribution | |  |  |  |  |  |  |  |  |  |  |  |  |  |
|  | Low risk | 225 (32.4) | 139 (29.5) | 123 (31.6) | 93 (25.5) | 107 (27.8) | 76 (23.0) | 53 (19.7) | 78 (26.3) | 49 (15.5) | 69 (18.0) | 39 (9.8) | 36 (9.2) | 40 (9.4) |
|  | Intermediate risk | 335 (48.3) | 245 (52.0) | 203 (52.2) | 187 (51.4) | 200 (51.9) | 180 (54.4) | 152 (56.5) | 132 (44.4) | 151 (47.8) | 184 (48.0) | 197 (49.2) | 196 (50.0) | 215 (50.2) |
|  | High risk | 134 (19.3) | 87 (18.5) | 63 (16.2) | 84 (23.1) | 78 (20.3) | 75 (22.6) | 64 (23.8) | 87 (29.3) | 116 (36.7) | 130 (34.0) | 164 (41.0) | 160 (40.8) | 173 (40.4) |
| Favorable localized PCa | | 255 (36.1) | 151 (31.9) | 115 (29.6) | 91 (24.9) | 103 (26.8) | 67 (20.3) | 41 (15.2) | 45 (15.2) | 44 (13.9) | 39 (10.2) | 12 (3.0) | 7 (1.8) | 7 (1.6) |
| Tumor stage at RP (≤pT2) | | 521 (73.6) | 345 (72.5) | 279 (71.5) | 252 (68.9) | 264 (68.2) | 208 (63.0) | 166 (61.7) | 192 (64.4) | 195 (61.5) | 231 (60.3) | 219 (54.6) | 246 (62.8) | 257 (60.1) |
| GGG of RP specimen | |  |  |  |  |  |  |  |  |  |  |  |  |  |
|  | GGG 1 | 312 (44.6) | 192 (40.7) | 143 (37.0) | 103 (28.2) | 117 (30.5) | 78 (23.8) | 49 (18.2) | 50 (16.9) | 50 (15.8) | 45 (12.0) | 14 (3.6) | 9 (2.3) | 7 (1.6) |
|  | GGG 2 | 162 (23.1) | 136 (28.8) | 135 (35.0) | 144 (39.5) | 157 (40.9) | 151 (46.2) | 130 (48.3) | 133 (44.9) | 134 (42.3) | 188 (50.3) | 180 (45.9) | 147 (37.9) | 171 (40.4) |
|  | GGG 2/3 | 60 (8.6) | 31 (6.6) | 13 (3.4) | 8 (2.2) | 3 (0.8) | 1 (0.3) | 0 | 1 (0.4) | 0 | 0 | 0 | 0 | 0 |
|  | GGG 3 | 68 (9.7) | 49 (10.4) | 48 (12.4) | 62 (17.0) | 53 (13.8) | 64 (19.6) | 56 (20.8) | 58 (19.6) | 63 (19.9) | 88 (23.5) | 113 (28.8) | 157 (40.5) | 167 (39.5) |
|  | GGG 4 | 53 (7.6) | 40 (8.4) | 18 (4.7) | 19 (5.2) | 16 (4.1) | 17 (5.2) | 18 (6.7) | 34 (11.4) | 36 (11.3) | 15 (4.0) | 18 (4.6) | 14 (3.6) | 21 (5.0) |
|  | GGG 5 | 45 (6.4) | 24 (5.1) | 29 (7.5) | 29 (7.9) | 38 (9.9) | 16 (4.9) | 16 (6.0) | 20 (6.8) | 34 (10.7) | 38 (10.2) | 67 (17.1) | 61 (15.7) | 57 (13.5) |
| PCa = prostate cancer; RP = radical prostatectomy; GGG = Gleason Grade Group | | | | | | | | | | | | | | |
